# Medication Discrepancies in Discharge Summaries and Associated Risk Factors for Elderly Patients with Many Drugs

**DOI:** 10.1007/s40801-019-00176-5

**Published:** 2019-12-13

**Authors:** Gabriella Caleres, Sara Modig, Patrik Midlöv, John Chalmers, Åsa Bondesson

**Affiliations:** 1 Department of Clinical Sciences in Malmö/Family Medicine, Center for Primary Health Care Research, Lund University, Box 50332, 20213 Malmö, Sweden; 2 Department of Medicines Management and Informatics in Skåne County, Kristianstad, Sweden; 3 The George Institute for Global Health, Sydney, NSW Australia

## Abstract

**Background and Objective:**

Elderly patients are at high risk for medication errors in care transitions. The discharge summary aims to counteract drug-related problems due to insufficient information transfer in care transitions, hence the accuracy of its medication information is of utmost importance. The purpose of this study was to describe the medication discrepancy rate and associated risk factors in discharge summaries for elderly patients.

**Methods:**

Pharmacists collected random samples of discharge summaries from ten hospitals in southern Sweden. Medication discrepancies, organisational, and patient- and care-specific factors were noted. Patients aged ≥ 75 years with five or more drugs were further included. Descriptive and logistic regression analyses were performed.

**Results:**

Discharge summaries for a total of 933 patients were included. Average age was 83.1 years, and 515 patients (55%) were women. Medication discrepancies were noted for 353 patients (38%) (mean 0.87 discrepancies per discharged patient, 95% confidence interval 0.76–0.98). Unintentional addition of a drug was the most common discrepancy type. Central nervous system drugs/analgesics were most commonly affected. Major risk factors for the presence of discrepancies were multi-dose drug dispensing (adjusted odds ratio 3.42, 95% confidence interval 2.48–4.74), an increasing number of drugs in the discharge summary (adjusted odds ratio 1.09, 95% confidence interval 1.05–1.13) and discharge from departments of surgery (adjusted odds ratio 2.96, 95% confidence interval 1.55–5.66). By contrast, an increasing number of drug changes reduced the odds of a discrepancy (adjusted odds ratio 0.93, 95% confidence interval 0.88–0.99).

**Conclusions:**

Medication discrepancies were common. In addition, we identified certain circumstances in which greater vigilance may be of considerable value for increased medication safety for elderly patients in care transitions.

## Key Points

**Table:** 

Medication discrepancies were frequently noted in discharge summaries for elderly patients with many drugs.
Discrepancies were more frequent in some situations, such as multi-dose drug dispensing and an increasing number of drugs, which may require further attention and preventive efforts.

## Introduction

Medication error is a failure in the treatment process with the potential to harm the patient [1]. According to the World Health Organization, medical errors and healthcare-related adverse events occur in about one in every ten hospitalisations [2]. Medication error may include the addition, withdrawal or changed dosage of a drug without documentation as stated by Midlöv et al. [3], while medication discrepancy is a wider concept commonly also including changes in the mode or frequency of administration [4, 5]. However, no clear and uniform definition exists [6].

Medication discrepancy and error rates in discharge documents vary across the world depending on the definition, method of measurement and selection of population. In Australian, American and Canadian studies, medication errors are noted in approximately 10–40% of discharge documents [7–9], while a literature review noted the occurrence of up to two errors per patient in discharge summaries [10]. Similarly, a Swedish study of elderly patients with many drugs showed, on average, two medication errors in every care transition [3]. Drug omission is the most common discrepancy and error type according to several studies [9–12]. The number of medications as well as increasing age seem to increase the discharge document medication discrepancy and error rate [11, 14].

A substantial proportion of all medication discrepancies and errors are potentially harmful [11–13, 15]. Considerable harm could be prevented, thus reducing health costs and patient suffering [2]. Preventing medication errors is heavily dependent on the discharge summary, which is commonly the only medication information a primary care patient receives after hospital discharge. Accordingly, its accuracy is crucial to enable smooth care transitions, but is often deficient according to primary care clinicians and previous studies [16, 17]. However, many studies include only a small sample of patients and have not focused solely on the elderly who are at a particular risk of re-admission and adverse outcomes after hospital discharge [18, 19]. The aim of this study was to examine the medication discrepancy rate and types for discharge summaries for elderly patients with many drugs, as well as the organisational, and care- and patient-specific factors affecting these errors.

## Methods

### Setting

The study was conducted in Skåne County, a region in southern Sweden where 1.3 million (13%) of the Swedish population lives [20]. The regions in Sweden are responsible for providing healthcare, while the smaller municipalities provide nursing care for the elderly. Physicians in the region collaborate with nurses from municipality care with regard to caring for elderly patients with medication aid from these nurses as well as nursing home residents. This region has ten hospitals of varying sizes and just over 150 primary healthcare centres. When a patient is discharged from the hospital, a discharge summary containing a medication report summarising which medication changes were made and why, as well as a medication list, is printed from the hospital electronic medical records and given to the patient and also sent by post to the primary care facility and the municipality [21]. The discharge summary also contains brief information about the cause of hospitalisation, what happened during the hospital stay and any plans after discharge. The discharge summary is partly electronically generated (data elements such as the medication list, admission and discharge dates, and patient name are automatically derived from the electronic medical records), while information such as what happened during the hospital stay and the medication report is written by a physician. Information transfer to the primary care facility also occurs via a medical case history; a more detailed document on the hospital stay without a medication list or report.

### Study Design

In this descriptive study, random samples of discharge summaries were collected by five pharmacists from 150 different departments (see Appendix) from all hospitals. To ensure consistent and reliable data collection and assessment, the pharmacists received training regarding the purpose and content of the discharge summary as well as information on current routines and guidelines. Multiple exercises on how to perform the data collection and interpretation followed. The pharmacists met regularly throughout the duration of the project and discussed and resolved any ambiguities. The departments were consecutively visited by the pharmacists. The eight last-written discharge summaries from the previous week, and the seven last-written discharge summaries from the week before last were collected. If there were fewer than 15 written discharge summaries during the preceding 2 weeks, the last 15 discharge summaries were collected.

The pharmacists performed a comprehensive retrospective medication history. First, an admission medication list was compiled as accurately as possible, by means of information from the multi-dose drug dispensing (MDD) system (a medication management assistance service with machine-dispensed disposable sachets in which medications are packaged according to the time of administration, common for elderly patients [22]) for patients using this system, and previous prescriptions as well as information from hospital electronic medical records on previous hospitalisations and outpatient care visits for all patients. Next, information on medication changes and adjustments from the current hospitalisation was retrieved from the hospital electronic medical records including the medication report in the discharge summary and prescriptions. Finally, a supposedly correct discharge medication list was compiled and compared to the medication information in the discharge summary to identify any medication discrepancies. We included the following customary medication discrepancies: omission or unintentional addition of a drug, incorrect daily dose, as well as incorrect details on mode and frequency of medication. In addition, the medication discrepancy noted as ‘missing information regarding temporarily stopped medications’ was also included if indicated as a major but not as a minor discrepancy by the pharmacist. ‘Major’ meant noted as temporarily stopped but should be taken. The discrepancy noted as ‘inheritance of previous prescriptions’ (i.e. adding the prescriptions in the electronic medical record from the patient’s last hospital stay) was equated with unintentional addition of a drug.

Organisational factors such as the department were also noted. Patient-specific factors noted were age, sex, number of drugs, MDD, discharge to home or nursing home, and whether any medication changes were performed during hospitalisation. Care-specific factors noted were physician medical training level, whether discharge occurred on a weekday or in the weekend, and whether a medication reconciliation (pharmacist identification of the most accurate medication list) or a team-based review (a structured and systematic review of the medication including risk assessment, adjustments and follow-up within a multi-professional team) was performed [23]. Pharmacist medication reconciliations and reviews are not routinely performed for all patients, but patients aged 75 years or older with five or more drugs are high priority. Criteria for further inclusion in our study were discharge summaries for patients aged 75 years or older with five or more drugs (continuous and as needed), collected from May 2015 until May 2016.

We aimed to answer the following questions:

- What is the medication discrepancy rate in the included discharge summaries?
- Which types of medication discrepancies are seen, and which substances and/or medication groups are involved?
- Which organisational and care- and patient-specific factors and substances and/or medication groups are associated with the medication discrepancies?

### Statistical Methods

Sample size was determined by the available sample, which was estimated as more than sufficient for the planned statistical analyses. Descriptive statistics was used to describe the patients and the medication discrepancies. Chi-square and *t* tests were used to compare groups. Univariate and multivariate logistic regression analyses were conducted to identify independent variables associated with the presence of any medication discrepancies. Assumptions for multivariate binary logistic regression were met. Linearity of the continuous variables with respect to the logit of the dependent variable was tested with a polynomial term and a Box-Tidwell (1962) test [24]. Based on this assessment, all continuous independent variables were found to be linearly related to the logit of the dependent variable. Because of the high correlation between the number of drugs according to pharmacist and the number of drugs in the discharge summary leading to multicollinearity, only the number of drugs in the discharge summary was included in the multivariate model. Negative binomial regression was conducted to assess the association between the independent variables and the medication discrepancy count.

### Ethical Considerations

The study was approved by the ethical committee in Lund (reference number 2018/404) as well as the regional advisory consultation group for quality control and healthcare data registers (reference number 191-18).

## Results

**Fig. 1:**
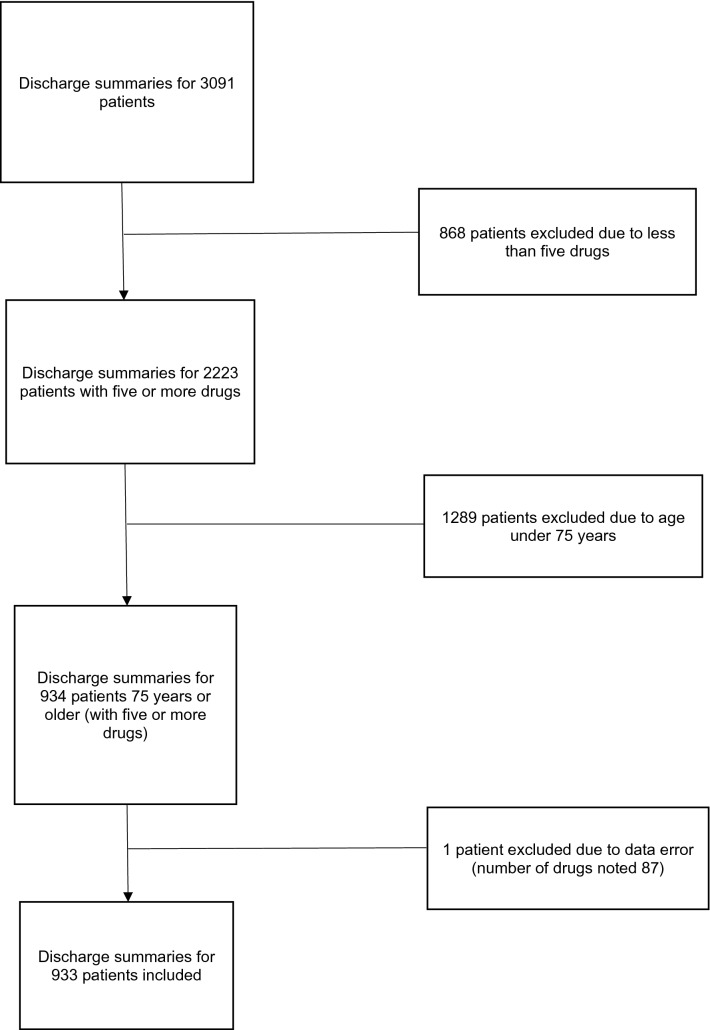
Inclusion flow chart

Discharge summaries for a total of 933 patients were included in the study (Fig. 1). Baseline characteristics are described in Table 1. The patients were discharged from nine hospitals (Landskrona 4%, Trelleborg 4.5%, Hässleholm 5%, Ängelholm 6%, Ystad 8%, Helsingborg 13%, Kristianstad 13.5%, Lund 21%, Malmö 25%, i.e. all hospitals in the region according to size but one of the smallest, Simrishamn), from departments of internal medicine (35%), surgery (18%), orthopaedics (12%), neurology (11%), geriatrics (8%), cardiology (7%), infectious diseases (5%), psychiatry (2%) and oncology (2%).

**Table 1:** Baseline characteristics for patients included in the study, in total and for patients with and without medication discrepancies (MD)

	All patients (*n* = 933)	Patients without MD (*n* = 580)	Patients with MD (*n* = 353)
Age	83.1 (75–100)	82.7 (75–99)	83.7 (75–100)
Women	515 (55%)	313 (54%)	202 (57%)
Men	418 (45%)	267 (46%)	151 (43%)
Medication reconciliation	180 (19%)	111 (19%)	69 (20%)
Team-based medication review during hospitalisation	146 (16%)	89 (15%)	57 (16%)
Number of on-demand use drugs^a^	2.3 (2.2–2.5)	2.1 (2–2.3)	2.7 (2.5–2.9)
Number of continuous use drugs	9 (8.8–9.3)	8.5 (8.2–8.7)	9.9 (9.5–10.3)
Number of all drugs^a^	11.4 (11.1–11.7)	10.6 (10.3–11)	12.6 (12.1–13.1)
Number of drug changes	3.2 (3–3.4)	3.3 (3.1–3.5)	3 (2.8–3.3)
Multi-dose drug dispensing^a^	250 (27%)	95 (16%)	155 (44%)
Discharging doctor			
Junior	387 (42%)	250 (43%)	137 (39%)
Resident	346 (37%)	210 (36%)	136 (39%)
Specialist	200 (21%)	120 (21%)	80 (22%)
Discharge on weekday	859 (92%)	532 (92%)	327 (93%)
Discharge on weekend	74 (8%)	48 (8%)	26 (7%)
Discharge to home	728 (78%)	478 (82%)	250 (71%)
Discharge to nursing home^a^	203 (22%)	102 (18%)	101 (29%)

The numbers are presented as totals and (%) apart from age, presented as mean (range) and number of drugs and drug changes, presented as mean (95% confidence interval)

^a^Statistically significant (*p* < 0.05) differences between groups

A total of 812 medication discrepancies were noted for all discharge summaries (mean 0.87 per discharge summary, 95% confidence interval (CI) 0.76–0.98), of which 530 were for continuous use drugs (mean 0.57, 95% CI 0.49–0.65) and 282 were for on-demand drugs (mean 0.30, 95% CI 0.25–0.35), respectively. The discrepancies were noted for 353/933 patients (38%), with a median of two discrepancies for these 353 patients (interquartile range 1–3). For 580 patients (62%), no discrepancies were noted. The discharge summaries contained 10,693 drugs in total, yielding a 7% error of all medications (95% CI 6.5–8.3). A comparison of baseline characteristics for patients with and without medication discrepancies is presented in Table 1.

Unintentional addition of a drug was the most common type of the 812 medication discrepancies (Table 2). Fifty-nine percent (206/347) of the unintentional additions of a drug were inherited previous prescriptions from a previous hospital stay.

**Table 2:** Types of all medication discrepancies noted in the discharge summaries, *n* (%)

	All drugs (*n* = 812)	Continuous use drugs (*n* = 530)	On-demand use drugs (*n* = 282)
Addition of a drug	347 (43)	224 (42)	123 (44)
Omission of a drug	268 (33)	150 (28)	118 (42)
Changes in dosage (lower/higher than intended)	146 (18)	108 (20)	38 (13)
Lacking information on temporarily stopped drugs	27 (3)	27 (5)	0
Change in frequency of administration	12 (1.5)	12 (2)	0
Change in mode of administration	12 (1.5)	9 (2)	3 (1)

The most commonly involved medication groups for all medication discrepancies according to the Anatomical Therapeutic Chemical classification system [25] main groups (first level), were Nervous system (229/812, 28%), Alimentary tract and metabolism (139/812, 17%) and Cardiovascular system (115/812, 14%) drugs. The most commonly involved therapeutic subgroups according to the second level of the Anatomical Therapeutic Chemical code were analgesics and drugs for constipation (Table 3). In total, the most commonly noted substances involved in the medication discrepancies according to the fifth level of the Anatomical Therapeutic Chemical code were paracetamol (74/812, 9%), oxycodone (39/812, 5%) and furosemide (37/812, 5%).

**Table 3:** Top ten most common therapeutic subgroups involved in the medication discrepancies (*n* = 812)

Therapeutic subgroup	*n*	%
Analgesics	150	19
Drugs for constipation	62	8
Psycholeptics	54	7
Drugs for obstructive airway disease	43	6
Diuretics	43	5
Emollients and protectives	33	4
Ophthalmologicals	29	4
Antithrombotic agents	29	4
Dermatological corticosteroids	27	3
Blood substitutes and perfusion solutions	26	3

The lowest proportion of discharge summaries with at least one medication discrepancy was noted for the Department of Geriatrics (18/72, 25%), while the highest proportions were noted for the Department of Infectious Medicine (21/43, 49%), Department of Oncology (8/17, 47%) and Department of Surgery (77/168, 46%). The univariate binary logistic regression analysis identified several significant risk factors for the presence of at least one medication discrepancy in the discharge summary (Table 4). In the final multi-variate model, MDD (adjusted odds ratio (OR) 3.42; 95% CI 2.48–4.74, *p* < 0.001) and an increasing number of drugs in the discharge summary (adjusted OR 1.09; 95% CI 1.05–1.13, *p* < 0.001) remained significant risk factors for the presence of at least one medication discrepancy, which was also noted for the Department of Surgery (adjusted OR 2.96; 95% CI 1.55–5.66, *p* = 0.001) and the Department of Oncology (adjusted OR 3.86; 95% CI 1.24–12.1, *p* = 0.02). An increasing number of drug changes was associated with a decreasing odds of medication discrepancy (adjusted OR 0.93; 95% CI 0.88–0.99, *p* = 0.017). The other independent variables were not significantly associated with the presence of at least one medication discrepancy in the multivariate models. Significant results from the negative binomial regression are presented in Table 5.

**Table 4:** Univariate binary logistic regression analysis of the risk factors for medication discrepancies

Characteristic	OR (95% CI)	*p* value
Multi-dose drug dispensing^a^	4.0 (2.95–5.42)	< 0.001
Discharge to nursing home^a^	1.89 (1.38–2.59)	< 0.001
Pharmacist medication reconciliation	1.03 (0.74–1.44)	0.878
Female sex	1.14 (0.87–1.49)	0.332
Discharge day weekend	0.89 (0.54–1.45)	0.618
Age	1.03 (1.01–1.06)	0.005
Number of drugs according to pharmacist^a^	1.10 (1.07–1.14)	< 0.001
Number of drugs in discharge summary^a^	1.11 (1.08–1.14)	< 0.001
Number of drug changes	0.96 (0.91–1.01)	0.123
Department of Surgery (reference category Geriatrics)^a^	2.54 (1.37–4.69)	0.003
Department of Internal Medicine	1.77 (0.99–3.15)	0.054
Department of Orthopaedics^a^	2.20 (1.15–4.23)	0.018
Department of Infectious Diseases^a^	2.86 (1.29–6.38)	0.010
Department of Cardiology	1.50 (0.72–3.14)	0.282
Department of Neurology	1.30 (0.66–2.55)	0.450
Department of Psychiatry	1.15 (0.36–3.69)	0.809
Department of Oncology	2.67 (0.90–7.94)	0.078
Discharging physician; resident (reference category junior)	1.18 (0.88–1.60)	0.275
Discharging physician; specialist	1.22 (0.86–1.73)	0.274
Team-based medication review	0.92 (0.39–2.15)	0.844

*CI* confidence interval, *OR* odds ratio

^a^Variables significantly affecting the OR of prevalence of medication discrepancy in the discharge summary

**Table 5:** Factors significantly associated with the number of medication discrepancies according to the negative binomial regression

Characteristic	OR (95% CI)	*p* value
Number of drugs in the discharge summary	1.08 (1.06–1.11)	< 0.001
Multi-dose drug dispensing	3.11 (2.34–4.14)	< 0.001
Department of Surgery (reference category Geriatrics)	3.77 (1.80–7.91)	< 0.001
Department of Orthopaedics	3.16 (1.45–6.88)	0.004
Department of Internal Medicine	2.30 (1.11–4.79)	0.026
Department of Infectious Diseases	3.70 (1.61–8.51)	0.002
Department of Oncology	5.02 (1.74–14.52)	0.003

*CI* confidence interval, *OR* odds ratio

## Discussion

We identified medication discrepancies in more than one third of the discharge summaries. Analgesics were the most commonly involved drugs, and unintentional addition of a drug was the most common discrepancy type. The strongest association with the presence and number of discrepancies was noted for MDD, an increasing number of drugs and discharge from departments of surgery.

The noted proportion of discharge summaries with medication discrepancies, with regard to the pharmacist-compiled admission medication list as well as medication changes and adjustments during hospitalisation according to hospital electronic medical records, were in accordance with an American study noting one or more discharge medication errors for 39% of the patients [7], as well as a Canadian study noting a matching discrepancy occurrence 30 days post-discharge [14]. A somewhat lower proportion of 29% of diabetic patients having at least one error at admission and/or discharge was noted in a French study [12]. In contrast, other European studies have shown considerably higher rates of medication discrepancies or inconsistencies in discharge documents [11, 26], and a recent Australian study noted one or more medication discrepancies in more than half of the electronic discharge summaries [15]. The noted medication discrepancy and error rate differences are possibly owing to varying definitions, study designs and settings. The average medication discrepancy rate in our study was nearly one in every discharge summary, similar to the discharge summary error rates (mean 0.81–1.42) noted by McMillan et al. [13]. The high proportion of medication discrepancies and errors noted in discharge documents are disquieting given the possible consequences such as therapeutic failure and adverse drug events [27], not to mention the risk of these documents losing their original intent and becoming less useful.

Whereas drug omission is a common medication discrepancy and error type [4, 12, 26, 28, 29], a greater proportion of unintentionally added drugs has also been previously noted [3] as was found in our study. The majority of these were inherited prescriptions from previous hospitalisations. Restarting a previously discontinued drug may result in unforeseen adverse consequences for the patient. A greater awareness of the most common cause of unintentionally added drugs may help prevent many discrepancies and errors. Drug omission was the second most common error type, in particular regarding on-demand use drugs, which is likely less serious than omission of a continuous use drug. However, omission of for example analgesics and diuretics when needed may also have a considerable impact on a patient’s health and well-being. In total, the highest number of discrepancies occurred for central nervous, alimentary tract and metabolism as well as cardiovascular drugs, consistent with findings in previous studies [3, 9, 30]. The most commonly affected medication group was analgesics, perhaps also the most common drugs for these patients. Analgesics have a high risk of side effects for elderly patients and need careful monitoring [31]. However, a lack of follow-up plans for analgesics for elderly patients in primary care was previously noted by our research group (unpublished data), which may reduce the chance of detecting any medication discrepancies.

Multi-dose drug dispensing was associated with a more than three-fold increased odds of medication discrepancies, consistent with a previous Swedish study on medication errors at discharge [3]. The importance of an adequate discharge summary medication list may be disputed for these patients because drug changes also should be carried out in the electronic MDD system. However, in a previous study by our research group, general practitioners stressed the lack of such changes being performed during hospitalisation [17]. Accordingly, these patients are at risk of having no accurate medication information, as are the general practitioners in primary care. Multi-dose drug dispensing, which is often used for elderly patients, is already known to be associated with a poorer drug quality and fewer drug changes [32, 33]. Our findings support the fact that MDD seems to be a clear risk area regarding drug safety for elderly patients despite its original intent. A lack of knowledge of the management of the MDD system has also previously been noted by the Swedish National Board of Health and Welfare [34], and training measures targeted at improving such inadequacies could be beneficial.

Furthermore, an increasing number of drugs was a significant risk factor for the presence as well as the number of medication discrepancies, as previously noted in many studies [11, 28, 35], not surprisingly given polypharmacy’s strong association with adverse drug events and drug-related problems [36, 37]. In contrast, no association between medication discrepancies and the number of medications was noted in a Singaporean study [4], although their patients only had half as many drugs as the patients in our study. However, in line with our results, no association with increasing age was noted, also in keeping with an American study that however showed a clear association between the number of medications and the number of discrepancies [37]. Hence, reducing polypharmacy may help counteract the occurrence of medication discrepancies. Therefore, continuously reassessing the drug therapy indication as well as considering non-pharmacological treatment alternatives and supplying good nursing care is of utmost importance [36]. Primary care routine follow-up (including medication reconciliation) after hospital discharge might be of value for the medication safety of patients with many drugs.

Discharge from a surgery department was associated with a nearly three-fold increased odds of medication discrepancies; contrary to a study from New Zealand showing fewer errors for general surgical service patients [13]. However, ‘surgery department’ in our study referred to a wide array of departments that may implicate differences in the study populations regarding for example age and number of drugs. The reason for the increased likelihood of discrepancies is not clear; possibly, surgeons focus more on surgery and its complications and hence less on medications and information transfer.

Further, we noted a decreasing risk and number of medication discrepancies for every medication change, unlike Salanitro et al. [7]. However, their study population was younger and included only patients with acute cardiac disease [7]. Many changes in medications may draw the physician’s attention to the medication list and thus increase its accuracy, such as by inherited prescriptions being noticed and ended.

Pharmacist medication reconciliation or team-based medication review during hospitalisation did not affect the likelihood of a discharge medication discrepancy in our study. However, improved discharge medication information as a result of pharmacist involvement has been noted in previous studies [37–39], mainly focusing on discharge interventions such as medication information preparation [38–40]. Such increased pharmacist involvement at discharge would likely be of importance for improved medication safety and deserves further attention.

Other factors showed no association with the presence of medication discrepancies, in line with for example, an Irish study showing no differences between discharge on a weekend or weekday [11], and an Australian study in which no impact of physician medical training level was noted [9].

### Strengths and Limitations

We examined a large study population of elderly patients with polypharmacy; a vulnerable group with a high risk of drug-related problems caused by medication errors and in great need of accurate discharge medication information in care transitions, the current state of which our study provides a good account. Furthermore, the pharmacists worked in close contact and collaboration with each other and data collection was performed in a structured manner, which guarantees a strong quality of the data. However, their inter-rater reliability was not assessed.

Further, the data did not include information on all drugs, why the proportion of discrepancies per medication group could not be described. Additionally, although the examined factors were relevant, some subgroups (such as some departments) tended to be small and some CIs were wide. Still, the main findings are solid, and we abstained from drawing any conclusions from results possibly affected by uncertainties regarding their interpretation. Further, we had no information on, for example, the length of hospital stay or diagnosis; other factors that may also be of importance. In addition, the accuracy of the medication *report* including reasons for changes was not assessed in this study, but the pharmacist also reviewed the hospital electronic medical records including prescriptions to assess the occurrence of any medication changes. Finally, overall, our study speaks merely of associations and not direct causal links; but points to where further attention and research could be directed.

### Future Research

While our study examined all medication discrepancies including those commonly referred to as errors, to further explore factors mainly associated with potentially harmful discrepancies, could help in pinpointing the efforts to enhance medication safety further. Additionally, factors only signalling an increased risk in the univariate analysis such as discharge to a nursing home may still be of interest and deserve further attention. More randomised controlled trials and interventional studies to further examine factors affecting the risk of medication discrepancies are also called for.

## Conclusions

Our study shows that discharge summary medication discrepancies are common, especially for patients with many drugs, using MDD or discharged from a surgery department. Awareness of these findings may increase caregivers’ vigilance and allow for targeted actions to prevent the generation of medication discrepancies, thus improving the quality of medication information in the discharge summaries and, by extension, improving medication safety for elderly patients in care transitions.

## Appendix

**Table:** Included departments

Main department	Additional departments
Surgery	Urology, breast surgery, surgical emergency, surgical gastrointestinal, plastic surgery, vascular surgery, ear, nose and throat, gynaecology
Orthopaedics	Hand surgery
Internal medicine	Internal medicine, emergency, endocrinology, renal, rheumatology, medical gastrointestinal, haematology, pulmonary medicine
Cardiology	
Infection medicine	
Neurology	Stroke, rehabilitation
Psychiatry	
Oncology	
Geriatrics	
